# Comparison of local sine wave modelling with harmonic phase analysis for the assessment of circumferential myocardial strain from tagged cardiovascular magnetic resonance images

**DOI:** 10.1186/1532-429X-14-S1-P277

**Published:** 2012-02-01

**Authors:** Christopher A Miller, Alexander Borg, David Clark, Christopher D Steadman, Gerry P McCann, Patrick Clarysse, Pierre Croisille, Matthias Schmitt

**Affiliations:** 1 University Hospital of South Manchester, Manchester, UK; 2 University of Manchester, Manchester, UK; 3 Alliance Medical, Wythenshawe CMR Unit, Manchester, UK; 4 NIHR Leicester Cardiovascular Biomedical Research Unit, Leicester, UK; 5 Université de Lyon, Lyon, France; 6 Université Jean Monnet, Saint-Etienne, France

## Background

Assessment of regional ventricular deformation is more sensitive than ejection fraction (EF) for detecting myocardial dysfunction. We sought to compare a local sine-wave modelling (SinMod) method with the more established harmonic phase analysis (HARP) technique, for assessment of Lagrangian left ventricular (LV) peak systolic circumferential strain (εcc) from tagged cardiovascular magnetic resonance images, in patients with cardiomyopathies and healthy volunteers. The variability and rapidity of each technique, and the effect of contrast, were also assessed.

## Methods

Sixty participants (15 each with hypertrophic, dilated or ischaemic cardiomyopathy and 15 healthy controls) with a wide range of LV ejection fraction (14-78%) underwent spatial modulation of magnetization tagging of a mid-ventricular short-axis slice at 1.5 Tesla. Global and segmental peak transmural εcc were measured using HARP and SinMod. Repeated measurements were performed on 15 randomly selected scans (25%) in order to assess observer variability. Tagged images were acquired pre- and post-contrast in 10 additional patients in order to assess the effect of contrast.

## Results

**Figure 1:**
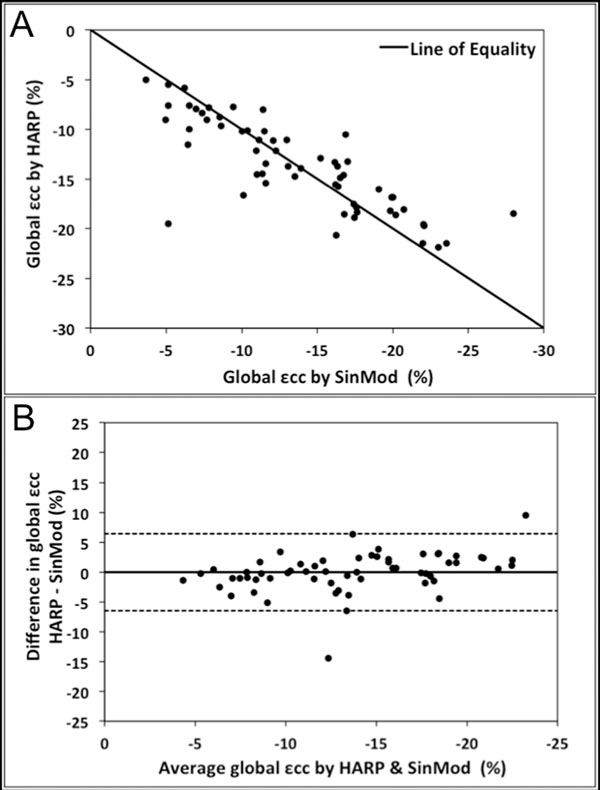
Agreement between local sine-wave modelling (SinMod) and harmonic phase (HARP) analysis methods for measurement of global peak systolic circumferential strain (εcc). (A) Scatter plot with line of equality; (B) Bland Altman plot.

**Figure 2:**
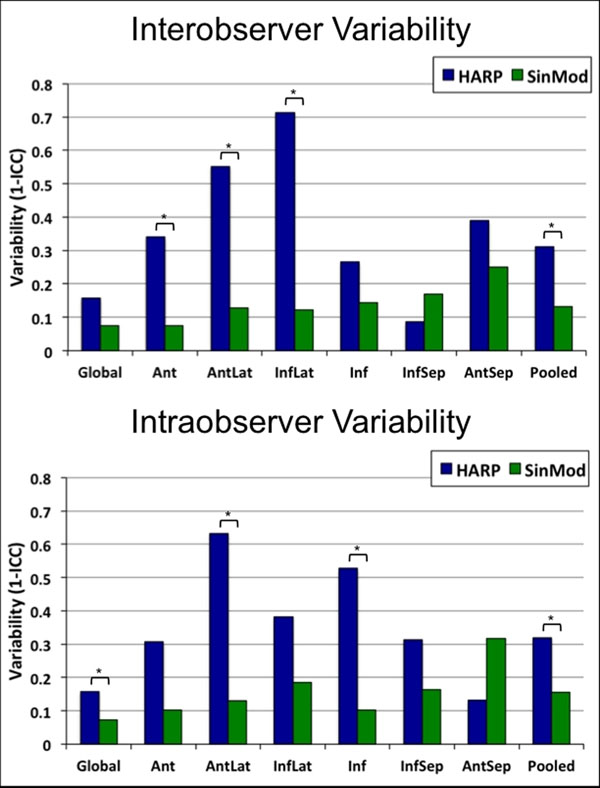
Interobserver and intraobserver variability for measurement of global and segmental peak systolic circumferential strain (εcc) using local sine-wave modelling (SinMod) and harmonic phase (HARP) analysis methods. Variability (calculated as 1 - intraclass correlation coefficient [ICC]) is greater for segmental εcc measurements than for global strain measurements using both techniques. SinMod had significantly lower intraobserver variability than HARP for measurement of global εcc. SinMod also had significantly lower inter- and intraobserver variability than HARP for pooled segmental εcc measurements. Ant - anterior segment, AntLat - anterolateral segment, InfLat - inferolateral segment, Inf - inferior segment, InfSep - inferoseptal segment, AntSep - anteroseptal segment, Pooled - pooled segmental analysis. * denotes a significant difference, assessed using a Wilcoxon rank comparison of the squared differences (p<0.05).

There was a high level of agreement between HARP and SinMod for global εcc (mean difference -0.02, 95% limits of agreement -6.46 to 6.43%, Figure 1). Agreement was much lower for segmental εcc, ranging from poor in lateral segments to modest in inferoseptal segments. Both methods showed excellent inter- and intraobserver agreement for global εcc (intraclass correlation coefficient>0.75). Inter- and intraobserver agreement for segmental εcc were also excellent with SinMod, and were significantly better than with HARP (p<0.0005, Figure 2). SinMod analysis time was significantly shorter than that for HARP (84±42 versus 201±120 seconds, p=0.02). Pre- and post-contrast global and segmental εcc measurements were not significantly different using either technique, although post-contrast measurements showed greater variability with HARP.

## Conclusions

SinMod and HARP-based measurements of global εcc have a high level of agreement. Agreement is substantially lower for measurement of segmental εcc. The SinMod method has generally lower observer variability, is faster and is less affected by contrast.

## Funding

Dr Miller was supported by a Doctoral Research Fellowship from the National Institute for Health Research, UK (NIHR-DRF-2010-03-98). Dr Schmitt was supported by Greater Manchester Comprehensive Local Research Network funding. Dr McCann and Dr Steadman received support from the NIHR Leicester Cardiovascular Biomedical Research Unit and additionally, Dr Steadman was funded by the British Heart Foundation (PG/07/068/2334).

